# TGFα-EGFR pathway in breast carcinogenesis, association with WWOX expression and estrogen activation

**DOI:** 10.1007/s13353-022-00690-3

**Published:** 2022-03-15

**Authors:** Karolina Pospiech, Magdalena Orzechowska, Magdalena Nowakowska, Dorota Anusewicz, Elżbieta Płuciennik, Katarzyna Kośla, Andrzej K. Bednarek

**Affiliations:** Department of Molecular Carcinogenesis, Medical University of Lodz, Lodz, Poland

**Keywords:** Estrogen receptor, Breast cancer, WW domain-containing oxidoreductase, nAnT-iCAGE, MCF7 cells, BT20 cells, MDA-MB-231 cells, T47D

## Abstract

WWOX is a tumor-suppressive steroid dehydrogenase, which relationship with hormone receptors was shown both in animal models and breast cancer patients. Herein, through nAnT-iCAGE high-throughput gene expression profiling, we studied the interplay of estrogen receptors and the WWOX in breast cancer cell lines (MCF7, T47D, MDA-MB-231, BT20) under estrogen stimulation and either introduction of the *WWOX* gene by retroviral transfection (MDA-MB-231, T47D) or silenced with shRNA (MCF7, BT20). Additionally, we evaluated the consequent biological characteristics by proliferation, apoptosis, invasion, and adhesion assays. TGFα-EGFR signaling was found to be significantly affected in all examined breast cancer cell lines in response to estrogen and strongly associated with the level of *WWOX* expression, especially in ER-positive MCF7 cells. Under the influence of 17β-estradiol presence, biological characteristics of the cell lines were also delineated. The study revealed modulation of adhesion, invasion, and apoptosis. The obtained results point at a complex role of the *WWOX* gene in the carcinogenesis of the breast tissue, which seems to be closely related to the presence of estrogen α and/or β receptors.

**Supplementary Information:**

The online version contains supplementary material available at 10.1007/s13353-022-00690-3.

## Introduction

Worldwide, breast cancer (BC) is not only the most often diagnosed tumor type among women, but also is the main causative of death due to oncological disorders (World Cancer Report et al. 2014). Data published in March 2019 by the Global Cancer Observatory (GLOBOCAN) show that there were 2 088 849 cases of breast cancer diagnosed, and 626 679 deaths due to this type of tumor in the year 2018 (Sharma 2021).

BC is a clinically and morphologically highly heterogeneous disease that differs in prognosis and response to treatment. Advances in the molecular and genetic field enhanced understanding of molecular pathways and genetic changes underlying breast cancer carcinogenesis, leading to more advanced and targeted BC management. One of the first breakthroughs in its treatment was made over a hundred years ago when it was found to be the hormone-dependent type of cancer (Beatson 1896). Indeed, up to now, hormone therapy is the oldest systemic management of BC. This phenomenon was for the first time associated with the presence of estrogen receptors in the late 1960s, upon their discovery (Toft and Gorski 1966; JENSEN 1962). Further studies of ER receptor confirmed not only its association with physiologic developmental and differentiation changes undergoing in the mammary gland but also with hormone sensitivity of breast cancer (Heldring et al. 2007).

ER receptors belong to a large family of nuclear receptors; however, for many years, it was believed that only one ER exists. In 1996, new estrogen-specific receptor was discovered, and was termed ERβ, and already known receptor ERα (Kuiper et al. 1996; Mosselman et al. 1996). Both have a similar structure, though are encoded by different genes—*ESR1* and *ESR2*—found at different chromosomal locations (chromosome 6 and 14, respectively). They also differ in tissue distributions, post-translational modifications, and cellular localization in healthy and malignant tissues (Burns and Korach 2012).

Currently, ERα is considered a key player in BC outgrowth and in developing anti-hormonal resistance. On the other hand, the exact role of ERβ in breast cancer is not yet fully understood; however, its discovery possibly explains ambiguous estrogen action in ERα-negative tissues.

Signal transduction through ER begins with binding with its ligand (estrogens), which results in a conformational change in receptor structure, enabling direct binding with specific DNA sequences, named estrogen response elements (ERE), promoting transcription of target genes. Apart from that classical pathway, ERs can interact with transcription factors Fos/Jun activating transcription at AP-1 sites (Kushner et al. 2000) or SP-1 at GC-rich (Sp1) promoter elements (Saville et al. 2000). What is interesting, unlike for ERE, signal transduction via AP-1 is opposite for both estrogen receptors—in case estradiol, ERα binding transcription activation is observed, whereas for ERβ, it is inhibited (Paech et al. 1997). Transcription of genes can be also influenced without ligand binding, through ER receptor phosphorylation (Heldring et al. 2007).

Estrogens are crucial for the development and maintenance of the reproductive system and proper sexual activity. But their biological range of action is much broader—encompasses both sexes and pertains to cardiovascular, musculoskeletal, immune, and central nervous systems (Heldring et al. 2007). Structurally, ligand-binding domains of both receptors display 53% homology but they differ only by two amino acid residues. This is sufficient for distinctive affinity towards various ligands (Abderrahman and Jordan 2018). Almost equal and very high affinity for ERα and ERβ was observed in the case of 17β-estradiol (E2). Other endogenous estrogens exhibit much lower affinity than estradiol, yet estrone (E1) binds preferentially to ERα, whereas estriol (E3) to ERβ (Kuiper et al. 1997).

ERα status is the most important predictor of breast cancer prognosis. ERα as a prognostic marker in BC is associated with increased survival in ERα-positive tumors, due to sensitivity to anti-estrogen therapy (Burns and Korach 2012).

The *WWOX* tumor suppressor gene coding region is located on chromosome 16q23.3–24, which is recognized as the one of the most common fragile sites of genetic change in breast cancer (Chen et al. 1996). Moreover, a decline in *WWOX* expression corresponds to shorter disease-free survival (DFS) and overall survival (OS) of BC patients (Guler et al. 2005), and gene was proposed as a potential prognostic marker in this tumor type (Pluciennik et al. 2010; Aldaz et al. 2014).

WWOX protein possesses enzymatic SDR (short-chain dehydrogenase/reductase) domain, with the coenzyme NAD(H)/NADP(H) binding site and two WW domains at the NH2 terminus (Bednarek et al. 2000, 2001). In Eukaryotes, proteins with WW domains are involved in numerous cellular processes, associated with cellular signaling, protein transport, transcription, and RNA processing. Indeed, for WWOX, there were over 40 partners identified, among them several transcription factors: such as P73 (Salah et al. 2010), AP2-gamma (Aqeilan et al. 2004a), c-Jun (Gaudio et al. 2006), DVL-2 (Bouteille et al. 2009), and RUNX2 (Aqeilan et al. 2008).

Analysis of the WWOX expression pattern in normal human tissues showed the highest expression in the testis, prostate, and ovary, and significantly lower in other tissues (Bednarek et al. 2000). In another wide range analysis, the highest expression was observed in the fallopian tubes, ovaries, mammary gland epithelial cells, endometrial, prostate, testes, liver, stomach, salivary glands, adrenal gland, thyroid, parathyroid, pituitary, cerebellum, and brain cells (Nunez et al. 2006). The highest expression observed in hormone-regulated tissues, additionally with the presence of an enzymatic dehydrogenase/reductase domain, suggested that its protein product may act as steroid dehydrogenase involved in the metabolism of steroid hormones (Bednarek et al. 2000; Duax and Ghosh 1997). Later on, the *WWOX* gene product was classified more precisely as 17β-hydroxysteroid dehydrogenase (Kallberg et al. 2002; Marijanovic et al. 2003). It was shown that WWOX fusion protein was active in the presence of both NAD + and NADP + towards steroid substrates such as 17β-estradiol, estrone, progesterone, and testosterone; however, its purification without losing activity has proved unsuccessful, suggesting that WWOX protein is in vivo present only in liaison with other cellular proteins (Saluda-Gorgul et al. 2011).

That WWOX protein participates in sex steroid metabolism, which was reinforced in animal studies (Chang et al. 2005). WWOX knocking-out in mice leads to deficiency in Leydig cell formation, untraceable levels of serum testosterone, decreased theca cell proliferation, and undersized ovarian follicles, which all point to the significant role of WWOX in steroidogenesis and proper gonadal function. In the same study, researchers revealed differential expression of 15 steroidogenesis-associated genes associated with WWOX absence (Aqeilan et al. 2009).

WWOX was correlated with estrogen receptor status in several studies. Immunohistochemical staining of archived BC specimens (*n* = 97) revealed reduced WWOX staining was more common in tumors with less favorable ER-negative (ER −) status (*p* = 0.033). Whereas 30.4% of cancer cases with normal WWOX staining showed negative or almost negative for estrogen receptors, 56.3% ER − tumors and less than 25% ER-positive (ER +) tumors exhibited low WWOX expression (Guler et al. 2009). Another study confirmed those results in a group of 16 human normal breast epithelium samples, 15 DCIS tumors, and 203 invasive breast cancer cases. A significant correlation (*p* = 0.0054) between WWOX level and ER status was observed, with the proportion of 46% ER − cases to 27% ER + breast carcinomas being negative for WWOX. When WWOX-deficient cases were analyzed together with nearly negative, the difference became more convincing (*p* = 0.003), with 73% of ER − cases to 51% of ER + cases (Nunez et al. 2005).

Level of *WWOX* expression correlated both with estrogen and progesterone receptor status in a study, where 132 breast cancer cases were evaluated utilizing quantitative real-time RT-PCR. The ER + tumors had *WWOX* expression several times higher than the ER − , which was also true for PR + cancers vs. PR − and ER + PR + tumors in relation to ER-PR − cases (Pluciennik et al. 2006). Positive and high relation of *WWOX* expression and with ER (*p* < 0.001) and PR (*p* = 0.001) was further confirmed by Guler G et al. through immunohistochemistry staining of tissue microarrays constructed from 837 breast cancer blocks (Guler et al. 2009).

Association of WWOX and hormone receptors was shown on an animal model as well. WWOX heterozygous C3H mammary tumor-susceptible mice (WWOX(C3H) + / −) exhibited loss of estrogen and progesterone receptors (Abdeen et al. 2011).

As we known, one allele of *WWOX* is deleted in more than 70% breast cancers and increasing WWOX expression in breast cancer cell lines reduce aggressiveness in animal xenografts. Therefore, we decided to find how WWOX expression differentiation affects global gene expression changes in association with estrogen.

Herein, we have tried to assess the relationship between the presence of estrogen receptors and the *WWOX* gene in breast cancer cell lines. The study was based on MCF7 and T47D estrogen-responsive cell lines, as well as MDA-MB-231 and BT20 estrogen-unresponsive cell lines, for which depending on native expression, the *WWOX* gene was either induced by retroviral transfection (MDA-MB-231, T47D) or silenced with shRNA (MCF7, BT20), and changes in the phenotype were estrogen-stimulated. We have employed nAnT-iCAGE methodology for high-throughput gene expression profiling.

MCF-7 breast cancer cell line is ERα-positive, which exhibits high proliferative potential under estrogen influence. Moreover, it shows one of the highest *WWOX* gene expressions among all studied breast cancer cell lines. On the other hand, BT-20 is recognized as an ERα-negative cell line, although estrogen receptor mRNA is expressed, but with exon 5 deletion (Castles et al. 1993). Additionally, *WWOX* expression for BT-20 is relatively high. T47D cell line possesses functional estrogen alpha receptor and exhibits a low expression of the investigated gene. Conversely, MDA-MB-231 cell line is well acknowledged as being ERα − , but ERβ + , and is an excellent model for *WWOX* gene studies for one of the lowest native expressions of this gene (Vladusic et al. 2000).

Under the influence of 17β-estradiol presence, we have also assessed biological characteristics of the cells, such as adhesion to extracellular matrix (ECM) proteins, invasion, proliferation, and apoptosis. These findings may implicate in the prediction of BC outcomes arising from biological consequences of the crosstalk between WWOX and ER during breast carcinogenesis. It also reveals new therapeutical opportunities due to identified aberrations of signaling pathways (e.g., EGFR) in response to ER signaling.

## Materials and methods

### Cell lines and culture conditions

T47D and MCF7 breast cancer cell lines were obtained from the American Type Culture Collection (ATCC), and grown according to the manufacturer’s protocol in RPMI-1640 Medium (Gibco) with 2 mM L-glutamine (Gibco), 10 mM HEPES, 1 mM sodium pyruvate, 4500 mg/L glucose, and 1500 mg/L sodium bicarbonate supplemented with 10% heat-inactivated fetal bovine serum (Gibco), antibiotics (Gibco, 0.05 mg/mL penicillin; 0.05 mg/mL streptomycin; 0.1 mg/mL neomycin), and human insulin (ITS insulin/transferrin/selenous acid Premix, BD Biosciences), in a humidified atmosphere containing 5% CO2 at 37 °C.

BT20 breast carcinoma cell line was obtained from CLS (Cell Lines Service GmbH, Eppelheim, Germany) and grown according to the manufacturer’s protocol in DMEM: Ham’s F12 medium (1:1 mixture) supplemented with 2 mM L-glutamine (Gibco), 10% heat-inactivated fetal bovine serum (Gibco), and antibiotics (Gibco, 0.05 mg/mL penicillin; 0.05 mg/mL streptomycin; 0.1 mg/mL neomycin) in a humidified atmosphere containing 5% CO2 at 37 °C.

Mammary gland adenocarcinoma cell line MDA-MB-231 was obtained from the American Type Culture Collection (ATCC) and was cultured in Advanced DMEM (Dulbecco’s modified Eagle’s medium) with 10% heat-inactivated fetal bovine serum, 4500 mg/L glucose, 2 mM L-glutamine, 1 mM sodium pyruvate, 10 mM HEPES buffer, and antibiotics (penicillin, 50 U/mL; streptomycin, 50 µg/mL; and neomycin, 100 µg/mL), in a humidified atmosphere of 5% CO2 at 37 °C.

For estrogen influence assays, cells were incubated for 48 h in above-listed culture media deprived of phenol red, supplemented with 10% charcoal stripped serum (Gibco). Cells were then treated either with 10^−8^ M 17β-estradiol (Sigma) or vehicle control (i.e., equivalent amounts of ethanol). Apart from untreated (time 0), samples were collected at 24 h after treatment.

### Stable retroviral transfection

The *WWOX* gene cDNA was introduced into T47D and MDA-MB-231 breast cancer cells via retroviral transfection, in which PT67 packaging line was used to produce the pLNCX2 retroviral vector with the cloned *WWOX* gene. T47D and MDA-MB-231 cells were grown up to 40% confluence and subsequently infected with 106 CFU/mL viruses suspended in culture medium supplemented with polybrene as a vehicle (8 μg/mL, Sigma-Aldrich). After 24-h incubation, stable transfectants were selected with 400 μg/mL G418 (Sigma-Aldrich) for 3 weeks, and transfection efficiency was confirmed by western blot analysis. A pool of stable transfectants was used for further biological experiments.

### Stable lentiviral transduction

MCF7 and BT20 breast cancer cells were plated in 24-wells plate at 4 × 104 cells in 1 mL growth medium 24 h prior to transduction. Subsequently cells were incubated in serum- and antibiotic-free media supplemented with polybrene (6 μg/mL and 4 μg/mL, respectively) with GIPZ lentiviral WWOX shRNA particles (Thermo Fisher Scientific, catalog no. VGH5523, clones ID: V2LHS_115633, V2LHS_255229 and V2LHS_411864), and GIPZ Non-silencing Lentiviral shRNA Control (catalog no. RHS4348) at MOI 5 (multiplicity of infection) for 24 h according to the manufacturer’s protocol.

Transduction efficiency was determined by the green fluorescence protein (GFP) positive cells, by means of fluorescence microscopy using a FLoid Cell Imaging Station (Life Technologies Corp.). Twenty-four hours after infection, 2 μg/mL puromycin (Life Technologies) was added for cell selection. Stable cell lines were obtained after 2 weeks. A pool of stable transductants was used for further experiments.

### Western blot analysis

For cell lysis and total protein extraction, RIPA protein extraction buffer supplemented with 1 mM phenylmethylsulfonyl fluoride (Sigma-Aldrich) and protease and phosphatase inhibitor cocktails (Sigma-Aldrich) was used. Protein concentration was determined by the Bradford method (Bio-Rad Laboratories). Sixty micrograms of extracted proteins was resolved on 10% SDS-PAGE (Bio-Rad Laboratories) and transferred on PVDF membranes (Sigma-Aldrich) by semi-dry blotting (Whatman; Biometra GmbH, Göttingen, Germany). The membranes were blocked for 1 h in 5% non-fat milk (Sigma-Aldrich) and incubated at 4 °C, overnight with a primary antibody (1:200 goat polyclonal anti-WWOX, Santa Cruz Biotechnology, cat. no. sc-20529). Subsequently, the membranes were washed with Tris-buffered saline-Tween 20 (TBST) buffer and incubated with the secondary antibody (1:15,000 anti-goat IgG, Sigma-Aldrich, cat. no. A4187) conjugated with alkaline phosphatase for 1 h at room temperature, followed by washes with TBST and developed with Novex® AP Chromogenic Substrate (Life Technologies). As a reference, mouse anti-GAPDG monoclonal antibody was used (1:1000; Santa Cruz Biotechnology, cat. no. sc-59540). The relative protein amount was assessed with ImageJ (Schneider et al. 2012) based on the integrated density of the bands.

### nAnT-iCAGE library preparation and sequencing

Libraries for next-generation sequencing (NGS) were prepared according to nAnT-iCAGE protocol (Murata et al. 2014). Briefly, 5 μg of total RNA was reverse transcribed using random N6 primer with 3-bp anchor and SuperScript III reverse transcriptase (Invitrogen), followed by oxidation of cap structure with sodium periodate (Sigma) and biotinylation with biotin (Long arm) hydrazide (Vector Laboratories). Subsequently, single-stranded RNA was digested with RNase I (Promega), whereas biotinylated RNA/cDNA duplexes were captured using magnetic streptavidin beads (Dynabeads® M-270 Streptavidin; Invitrogen). Unbound RNA/cDNA hybrid molecules were washed away, and single-stranded cDNA released by treatment with RNase H (Invitrogen), RNase I (Promega), and heat, followed by both end linker ligation—barcoded 5′ linker and 3′ linker. Eight different barcodes were used in the procedure. Second strand was synthesized by nAnT-iCAGE 2nd primer, which anneals to 5′ linker. Final products were assessed for their quality by means of Agilent Bioanalyzer High Sensitivity DNA kit (Agilent Technologies) and their concentration by Quant-iT™ PicoGreen® dsDNA Kit (Invitrogen). Five ng libraries were sequenced on NextSeq 550 Illumina sequencer and NextSeq 500/550 High Output Kit, 75 reads. Samples for cluster generation were prepared according to (CAGE^TM^ Preparation Kit n.d.).

### nAnT-iCAGE data processing and expression analysis

Processing and analysis of sequenced nAnT-iCAGE data was done with the workflow proposed by the Data Analysis Center of the Cell Innovation Program (https://cell-innovation.nig.ac.jp/maser/Applications/CAGE-seq_en.html), employing Galaxy—an open source, web-based platform (Afgan et al. 2018). Briefly, FASTQ sequenced data files were first assessed for proper quality by FastQC tool (Andrews n.d.) and split by a barcode sequence by means of Barcode Splitter tool (Gordon n.d.). Subsequently, AfterQC tool was used for filtering, barcode trimming, error removal, and quality check of fastq files (Chen et al. 2017). Burrows-Wheeler Alignment tool (BWA) (Li and Durbin 2009) was used for reads alignment and mapping to the human reference genome (GRCh38). The data were recorded in SAM (Sequence Alignment/Map) format, transformed to BAM (Binary Sequence Alignment Map) files and alignments sorted by means of SAMtools package (Li et al. 2009a). CAGEr R package (Haberle et al. 2015) was employed for identification of transcription start sites (TSS) and their usage frequency, raw tag count normalization, and construction of promoterome. Full genome sequences for human “BSgenome.Hsapiens.UCSC.hg38” were used (Team 2020). For each experimental point, at least 1.5 × 10^−6^ transcripts mapped to the human genome were obtained. Finally, gene expression is shown as TPM (transcript counts per million transcripts mapped). Statistical analysis of differentially expressed genes was performed using NOIseq algorithm (Tarazona et al. 2015).

ERα and ERβ target gene list was downloaded from Harmonizome database (Rouillard et al. 2016). For ERα, 1791 ENCODE-verified target genes were analyzed and for ERβ, it was 419 ChEA-verified target genes. Differential expression between experimental points was assayed using UpSetR software (Conway et al. 2017). Gene ontology analysis was performed with ShinyGO web tool at (Ge et al. 2020).

### Proliferation and apoptosis assay

A triplex test simultaneously evaluating cellular redox potential (data not shown), proliferation (data not shown), and apoptosis of the cells was used in order to reduce population and systematic discrepancies between cell cultures. Apoptosis was assessed with the DELFIA® DNA fragmentation assay (PerkinElmer), which exploit terminal deoxynucleotide transferase dUTP nick end labeling (TUNEL reaction) and samarium-labeled streptavidin.

Ten thousand cells/well were incubated for 48 h in culture media deprived of phenol red, supplemented with 10% charcoal stripped serum (Gibco) on a white, clear bottom 96-well plates. Cells were then treated either with 10^−8^ M 17β-estradiol (Sigma) or vehicle control (i.e., equivalent amounts of ethanol). Samples were collected at time 0 (untreated) and 24 h after treatment.

### Adhesion assay

Adhesion assay evaluating capability of cells to adhere to four extracellular matrix (ECM) proteins—fibronectin, collagen I, collagen IV, and laminin—was carried out in order to assess the ability of the cells to integrate into the ECM. After 48-h incubation of cells in culture media deprived of estrogens (without phenol red, supplemented with 10% charcoal stripped serum) and subsequent treatment with 10^−8^ M 17β-estradiol (Sigma) or vehicle control, the cells were seeded in serum free media at the density of 300 000 cells/well on the 24-well plates coated with selected ECM proteins and uncoated control (Corning® Biocoat™ 24 well clear Flat Bottom TC-Treated Multiwell Plates; cat. no. 354411, 354,408, 354,430, 354,412) and allowed to adhere for 90 min at 37 °C. Adherent cells were stained with 0.09% crystal violet, the stain extracted with 10% acetic acid and quantified colorimetrically.

### Invasion assay

The invasive potential of the investigated cells was evaluated using the colorimetric CytoSelect™ 24-well invasion assay (Cell Biolabs, Inc.). The assay contains a membrane coated with a layer of basement membrane matrix solution and allows for discrimination of invasive cells. The cells were seeded on inserts placed in a 24-well plate at a density of 300 000 cells/well and left to invade for 48 h (according to the manufacturer’s protocol). Next, the cells that crossed the membrane were dyed and their number was analyzed colorimetrically.

### Statistical analysis

Data was presented as the means ± standard error. Statistical significance between the samples was assessed using the Aspin-Welch *t* test and recognized as being statistically significant at a confidence level > 95% (*p* < 0.05). All the statistical analyses were performed using Statistica 12 software (StatSoft Inc.).

## Results

### WWOX expression evaluation

**Fig. 1:**
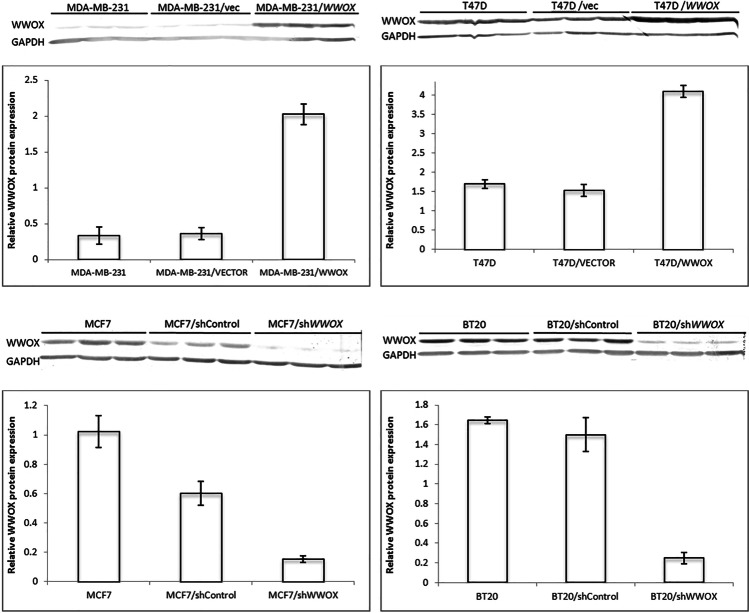
Relative WWOX protein expression levels after upregulation by retroviral transfection (top panel) and downregulation by lentiviral transduction (bottom panel). T47D and MDA-MB-231 cell lines were transduced using retroviral vector carrying *WWOX* cDNA. MCF7 and BT20 wild-type cells showing high WWOX expression were transduced using lentiviral vector harboring WWOX shRNA. More detailed description may be found in the “Materials and methods” section

The successfulness of WWOX protein level down- and upregulation was confirmed by western blot analysis before any assays (Fig. 1). The increase in relative WWOX protein expression for T47D and MDA-MB-231 cell lines was 2.68 and 5.58, respectively, whereas a decrease of WWOX protein expression for MCF7 and BT20 cell lines was 3.89 and 6.00, respectively.

### WWOX-ER interplay in adhesion, invasion, and apoptosis

All the cell variants, for which stable modulation of WWOX expression was observed, were treated with 17β-estradiol (or equivalent amounts of ethanol, further in text denoted as VC, standing for vehicle control) in time points (*t* = 0 h, *t* = 24 h) and sequenced. Due to low sequencing quality for T47D, this cell line was removed from bioinformatic analyses following biological assays.

### Adhesion

**Fig. 2:**
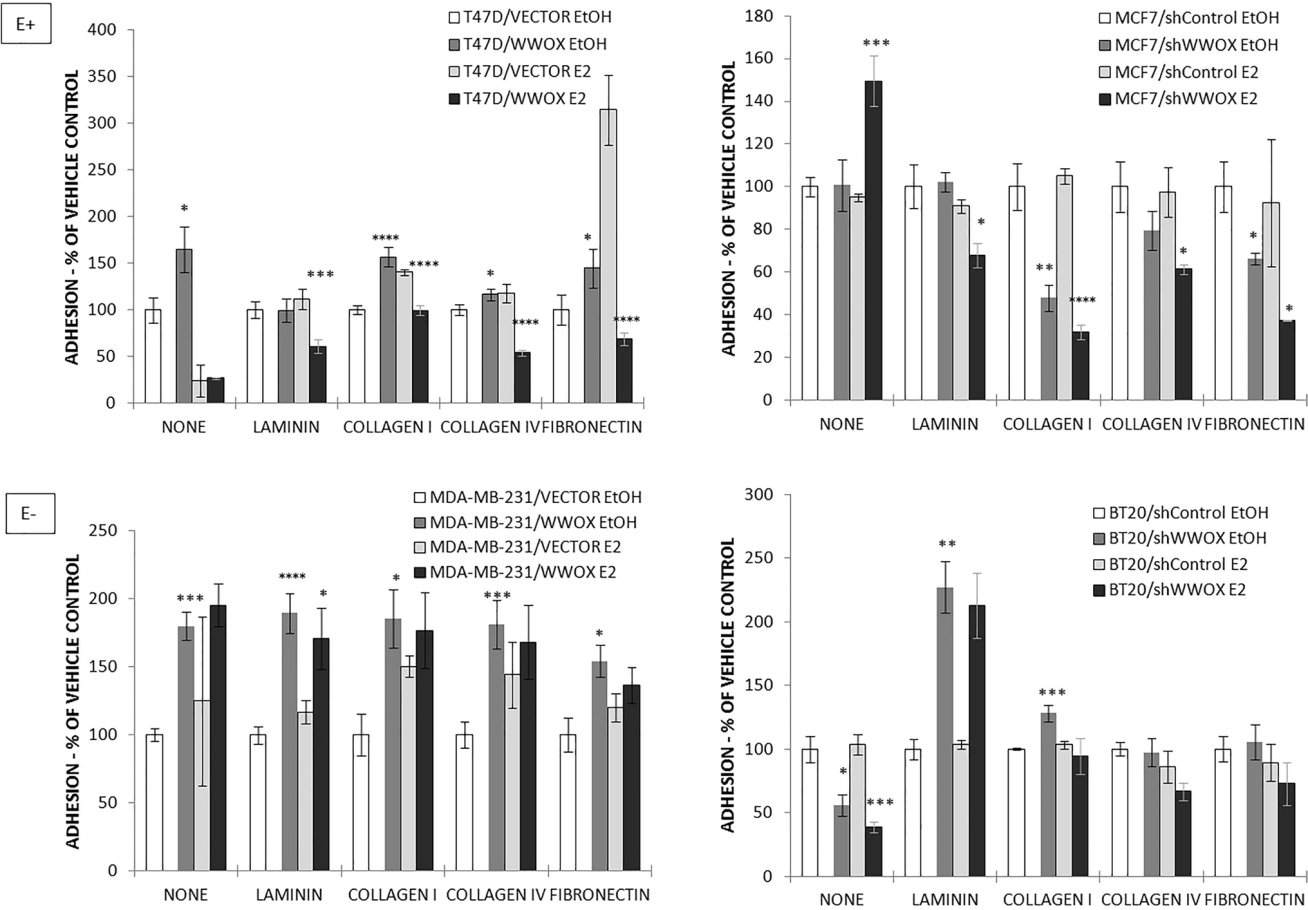
The adhesion characteristics of breast cancer cell lines, with respect to *WWOX* gene expression and estrogen influence. As expected, most substantial changes after estrogen treatment (E +) or vehicle control (E −) were observed for estrogen-responsive cell lines T47D and MCF7, where for all investigated ECM proteins, significant adhesion differences were noticed. Key: *p ≤ 0.05, **p ≤ 0.01, ***p ≤ 0.005, ****p ≤ 0.001

As mentioned above, to confirm NGS data, adhesion to ECM proteins was assessed biologically, by means of four different ECM components—laminin, collagen I, collagen IV, and fibronectin (Fig. 2).

For the T47D cell line, after *WWOX* upregulation, most significant changes were observed in adhesive properties to collagen I (1.56-fold increase, *p* = 0.001) and fibronectin (1.44-fold increase, *p* = 0.043). A slight increase was also observed for collagen IV (1.16-fold, *p* = 0.026). Similarly, overexpression of *WWOX* gene in MDA-MB-231 cell line resulted in increased adhesion to all investigated ECM proteins (laminin, 1.89-fold, *p* = 0.001; collagen I, 1.85-fold, *p* = 0.017; collagen IV, 1.81-fold, *p* = 0.002, and fibronectin, 1.54-fold, *p* = 0.005). In MCF7 cell line, *WWOX* gene silencing reverted the relation—a decrease was observed for collagen I (2.09-fold, *p* = 0.010), collagen IV (1.26-fold, *p* = NS), and fibronectin (1.51-fold, *p* = 0.014). Interestingly, for BT20 cell line, *WWOX* downregulation significantly increased adhesive properties of the cells to laminin (2.27-fold, *p* = 0.002) and collagen I (1.28-fold, *p* = 0.002). All above given results are summarized in Supplementary Table 1 available at GitHub repository (https://github.com/orzechmag/wwox-er), along with the other supplementary materials.

The greatest changes in adhesive properties upon estrogen treatment after *WWOX* gene transduction were observed for estrogen-responsive cell lines, for which a significant decrease in all of ECM proteins was detected. For T47D cell line, we have observed decrease in adhesion to laminin (1.83-fold, *p* = 0.003), collagen I (1.41-fold, *p* = 3.0 × 10^−4^), collagen IV (2.19-fold, *p* = 4.6 × 10^−4^), and fibronectin (3.85-fold, *p* = 0.019), whereas for MCF7 cell line, it was for laminin (1.34-fold, *p* = 0.015), collagen I (3.29-fold, *p* = 1.4 × 10^−5^), collagen IV (1.59-fold, *p* = 0.03), and fibronectin (2.48-fold, *p* = 0.04) (Supplementary Table 2, Fig. 2).

Contrastingly for estrogen-unresponsive MDA-MB-231 cell line, *WWOX* gene transduction increased adhesion after 17β-estradiol treatment for all investigated proteins, but statistically significantly were only for laminin (1.46-fold, *p* = 0.017). For estrogen-unresponsive BT20 cells, no significant changes in adhesion to ECM proteins were observed (Supplementary Table 2, Fig. 2).

### Invasion

**Fig. 3:**
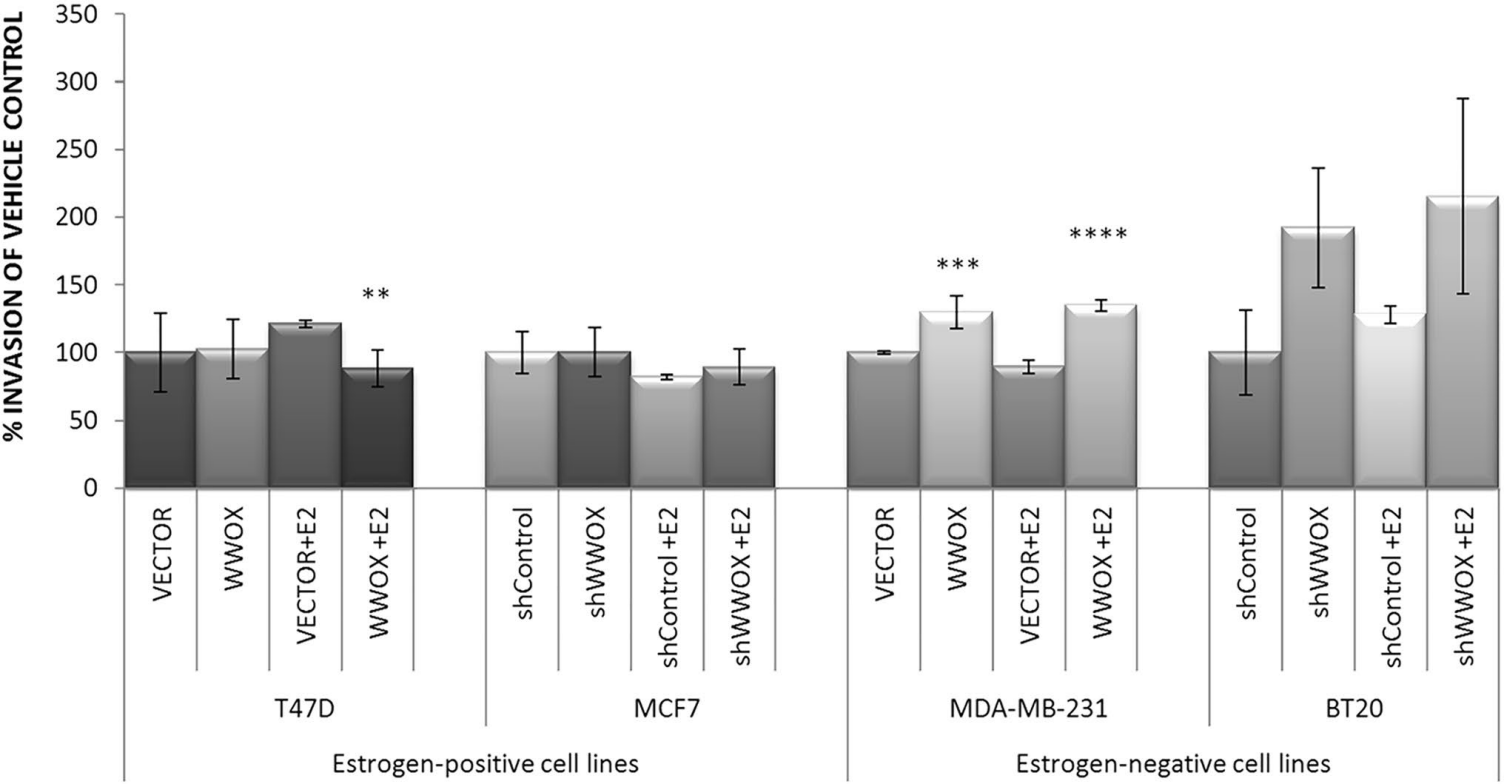
The invasiveness of breast cancer cell lines, with respect to *WWOX* gene expression and estrogen influence. The assay was conducted using basement membrane proteins coated inserts which create a barrier that can be passed by invasive cells (see the “Material and methods” section). Key: *p ≤ 0.05, **p ≤ 0.01, ***p ≤ 0.005, ****p ≤ 0.001

The epithelial-to-mesenchymal transition (EMT) is considered a crucial event in gaining by the cells’ invasive potential. Therefore, the invasive potential of the investigated cells was evaluated biologically by measuring the number of cells invading across the basement membrane matrix solution (Fig. 3).

In estrogen-positive cell lines—T47D and MCF7—*WWOX* expression modulation did not affect the invasiveness of the cells. Instead, the MDA-MB-231 cell line after *WWOX* gene restoration exhibited a 1.3-fold increase in invasive potential (*p* = 0.0026).

In the estrogen-responsive T47D cell line, estrogen exposure and *WWOX* upregulation resulted in a 1.4-fold decrease of invasion (*p* = 0.0058), whereas in the estrogen-negative MDA-MB-231 cell line, 1.5-fold increase (*p* = 8 × 10^−6^) in invasion was observed.

### Apoptosis

As for adhesion and invasion, also apoptosis was evaluated in biological experiments, by measuring the degree of DNA fragmentation (DELFIA® DNA fragmentation assay, PerkinElmer). It was part of a triplex test, which additionally evaluated redox potential and proliferation (data not shown), but did not return any significant findings.

**Fig. 4:**
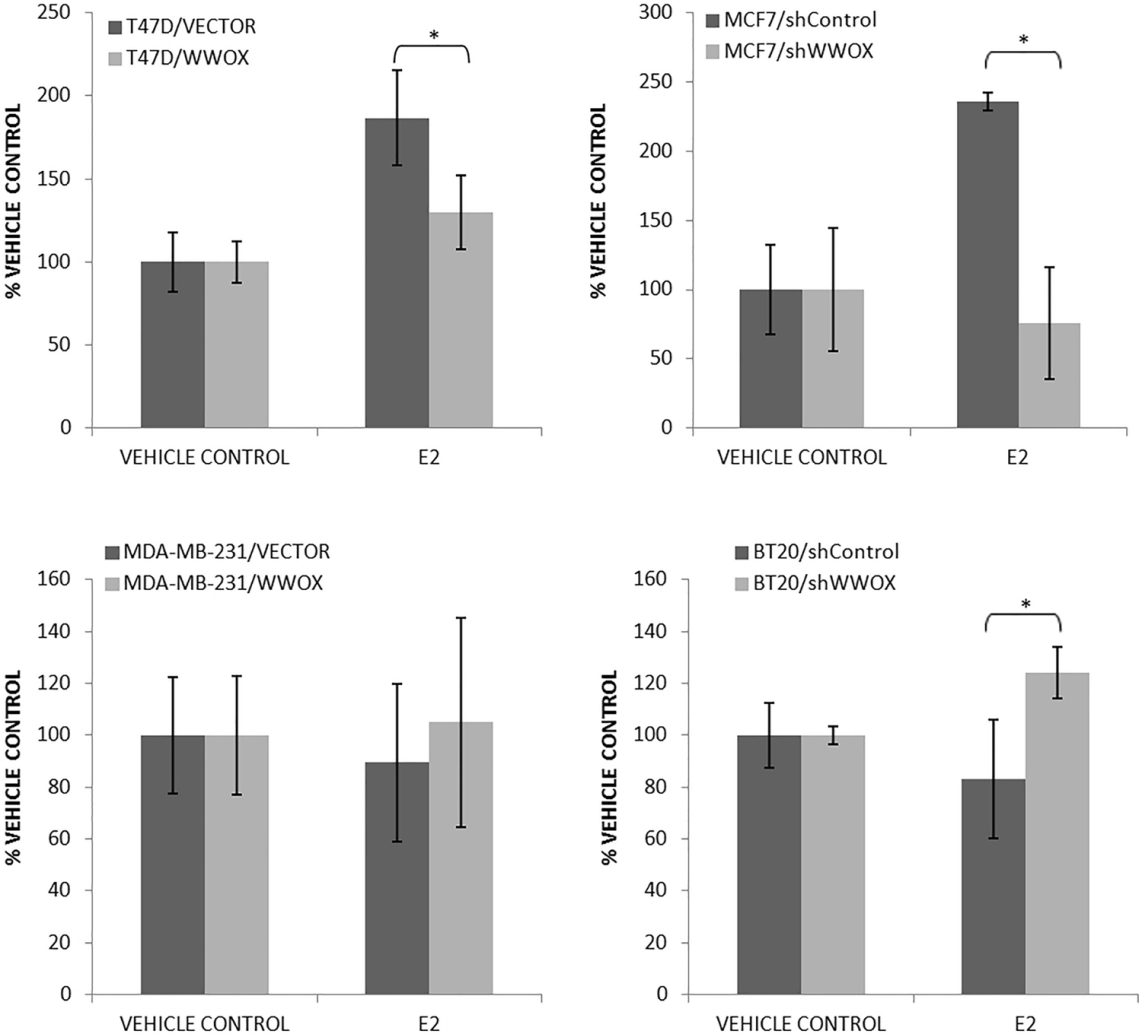
Apoptosis in breast cancer cell lines with respect to *WWOX* gene influence and estrogen treatment. The graph shows increase in apoptosis relative to the control transductions with (E2) and without (vehicle control) estradiol treatment. Key: *p ≤ 0.05, **p ≤ 0.01, ***p ≤ 0.005, ****p ≤ 0.001

On the other hand, we have observed substantial changes in apoptotic potential of cell lines under investigation, both as a result of differential *WWOX* gene expression and as simultaneous with the transfection estrogen treatment (Fig. 4).

The results of apoptosis after 24-h treatment with estradiol for investigated transfection variants are equally interesting (Fig. 4). As a result of estrogen treatment, there was a statistically significant decrease in apoptosis in both estrogen-dependent cell lines, T47D and MCF7 (1.44-fold, *p* = 0.0201, and 3.11-fold, *p* = 0.0133, respectively), and an increase in apoptosis for estrogen-independent breast cancer cells, MDA-MB-231 and BT20, though only for the latter, the change turned out to be statistically significant (1.49-fold, *p* = 0.0173).

### Differential expression of ERα and ERβ target genes associated with WWOX silencing

Differential expression CAGE analysis revealed 13,076 genes expressed in three cell lines MCF7, BT20, and MDA-MB-231. For differential gene expression (DGE) analysis in BT20 and MDA-MB-231 cell lines, we excluded genes showing very low expression, and analysis was limited only to genes that showed TPM above five in any cell line analyzed in any point measured. To find expression differences between WWOX protein level and E2 treatment, we compared fold change (FC) of gene expression between a cell with low versus high WWOX protein level according to estradiol (E2) treatment versus vehicle only. Table 1 shows the number of *ESR1* and *ESR2* genes which expression was identified in assayed cell lines.

**Table 1:** The total number of ERα and ERβ target genes identified via database corroborated with the number of targets identified in assayed cell lines the TPM > 5

	Database	MCF7	BT20	MDA-MB-231
ERα targets total	1791			
ERα targets TPM > 5		902	896	817
ERβ targets total	419			
ERβ targets TPM > 5		278	253	224
ERα and ERβ targets total	103			
ERα and ERβ targets TPM > 5		68	65	53

**Fig. 5:**
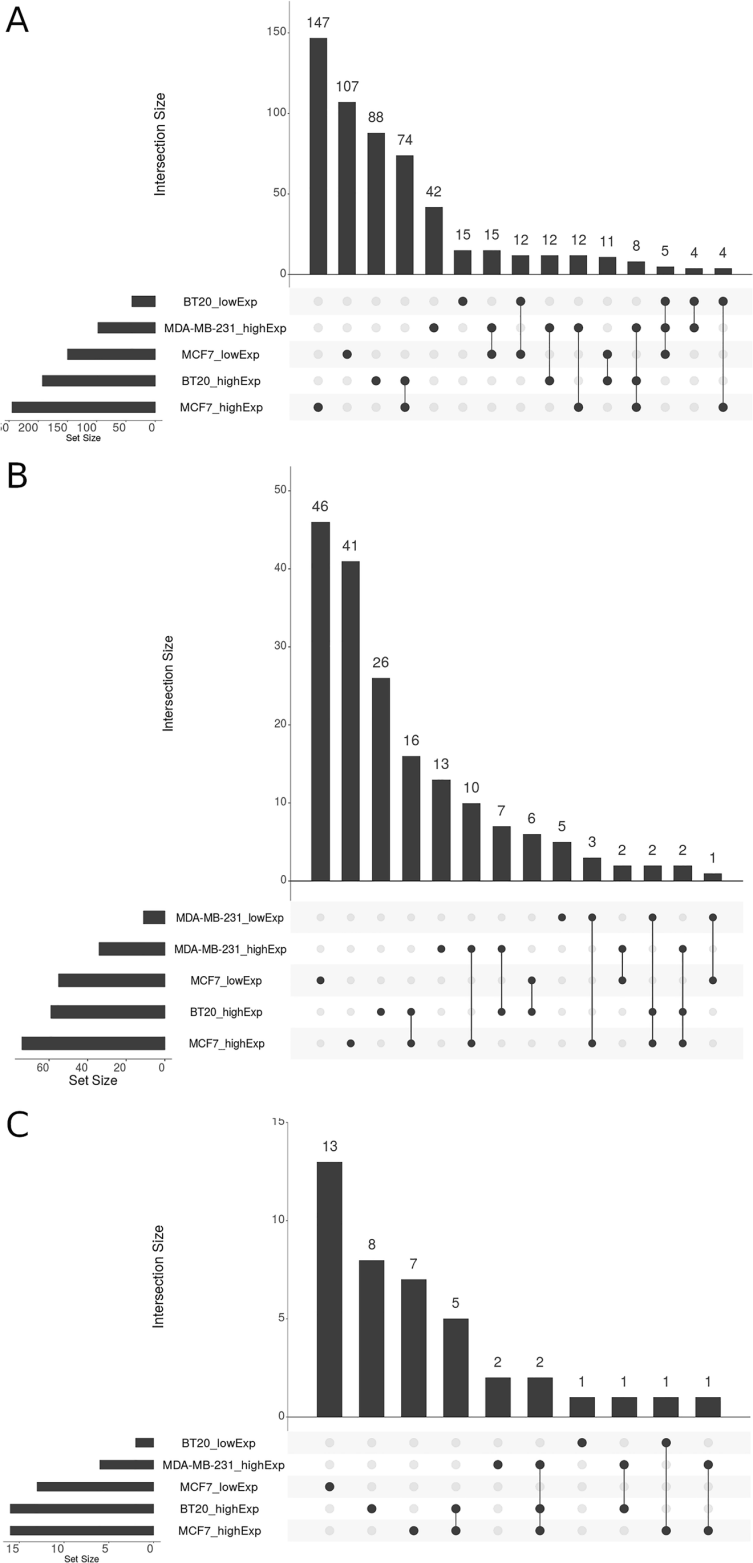
Graphs show UpSetR analysis of mutually exclusive expression or co-expression genes regulated by estrogen receptors according to databases ENCODE for ERα, ChEA for ERβ, or both. Analysis was performed only for genes which calculated expression was not lower than TPM = 5 (at least at one measurement point) and expression fold change was bigger than 2 after 24-h estradiol treatment in cells showing low *WWOX* expression relative to high *WWOX* expression. Intersection size bar numbers represent a number of genes co-expressed or showing mutually exclusive expression between examined cells. In cell lines, names lowExp and highExp mean lowered or elevated expression respectively

Supplementary Table 3 contains a list of all 13,076 transcripts identified in examined cell lines with their TPM values as well as ENCODE and ChEA ERα and ERβ target genes and their TPM expression numbers and TMP > 5 filtered and fold change values used in this analysis. Figure 5 shows analysis of ERα and ERβ target genes showing lowered or elevated (FC > 2) expression in cell line constructs with low versus high expression of *WWOX* after 24-h estradiol treatment. As expected, the highest expression differentiation was found in an estrogen-dependent MCF7 cell line with 147 genes expressed higher in MCF7 WWOXlow versus MCF7 WWOXhigh and 107 genes showing lowered expression respectively. A relatively low number of target genes showed differential expression in two or three different constructs (Fig. 5A). A similar effect was found for ERβ and both estrogen receptors where the largest differential expression was also found in MCF7 cells (Fig. 5B and C). Lists of estrogen target genes showing mutually exclusive expression or co-expression in MCF7, BT20, and MDA-MB-231 cell lines according to WWOX protein level (FC change WWOXlow vs WWOXhigh) after 24-h E2 treatment is enclosed as Supplementary Tables 4 (*ESR1*), 5 (*ESR2*), and 6 (*ESR1a2*).

Gene ontology analysis returned that most ERα target genes which estradiol differentiated expression was affected by WWOX protein can be divided into four groups according to Biological Processes ShinyGO classification: cell death, cell adhesion, signal transduction, and metabolism regulation.

The transcription of many genes is regulated by more than one transcription factor. Using ShinyGO analysis, we identified 29 TFs that co-regulate ERα target genes (FDR < 0.05). Supplementary Table 7 lists transcription factors and ERα co-targeted genes. The function of three of them can be regulated by *WWOX*: *CREB1* and *MYC* directly and *FOXA1* through *TFAP2C-WWOX* integration (Woodfield et al. 2010; Li et al. 2009b; Khawaled et al. 2019). According to our findings, *CREB1* and ERα co-regulate expression of 329 genes identified in MCF7, *FOXA1* 309 genes, and MYC 381 genes. Interestingly, we identified 40 *CREB1-ERβ* co-targeted genes differentially expressed according to WWOX protein level in MDA-MB-231 cell line which is known to express *ESR2* receptor (Vladusic et al. 2000).

In MCF7 cells, we found differential expression of 98 genes classified by GO to regulation of signaling (Supplementary Table 8). One of them is survivin (*BIRC5*) upregulated in MCF7 WWOXlow under E2 treatment. Some other genes crucial for cancer metabolism identified in regulation of signaling set (GO:0,023,051) are *ERBB2*, *TGFA*, *GLI3*, *MUC1*, *BAD*, *BCL6*, *CASP4*, *JDP2*, *ROBO1*, and *HES1.*

Our results show that *WWOX* differential expression together with the effects of estrogen significantly affects gene expression profiles of model breast cancer cell lines. We observed association of *WWOX* with expression changes of genes directly regulated by estrogen receptors. However, these changes also involve a number of signaling pathways and transcription factors which consequently indirectly regulates more genes. As a result, we observe biological changes important for the development and progression of the neoplastic disease, such as proliferation, apoptosis, adhesion, and invasion.

## Discussion

In the study, we performed biological and global gene expression analysis of breast cancer cell lines in the context of differential expression of *WWOX* tumor suppressor gene in association with estrogen receptor response. Numerous reports are showing *WWOX* as a critical player in breast carcinogenesis and progression. There are also several studies showing the association of *WWOX* expression with ER status of breast cancer and cancer invasiveness. We show here changes in cell viability, apoptosis, invasion, and ECM adhesion of ER-positive and ER-negative breast cancer cell lines supporting this data with analysis of global gene expression profiles.

In the study, we have used NGS CAGE to reveal the relation between estrogen receptor and *WWOX*, which might shed some light on its function and significance in hormone-related tissues. We have assayed ERα (*ESR1*) and ERβ (*ESR2*) target genes according to WWOX low and high expression and estradiol treatment in breast cancer cell lines estrogen receptor alpha (ERα)-positive MCF7 which has native high *WWOX* gene expression in comparison with BT20 ERα-negative and high *WWOX* and MDA-MB-231 which is ERα-negative with low *WWOX* expression.

**Fig. 6:**
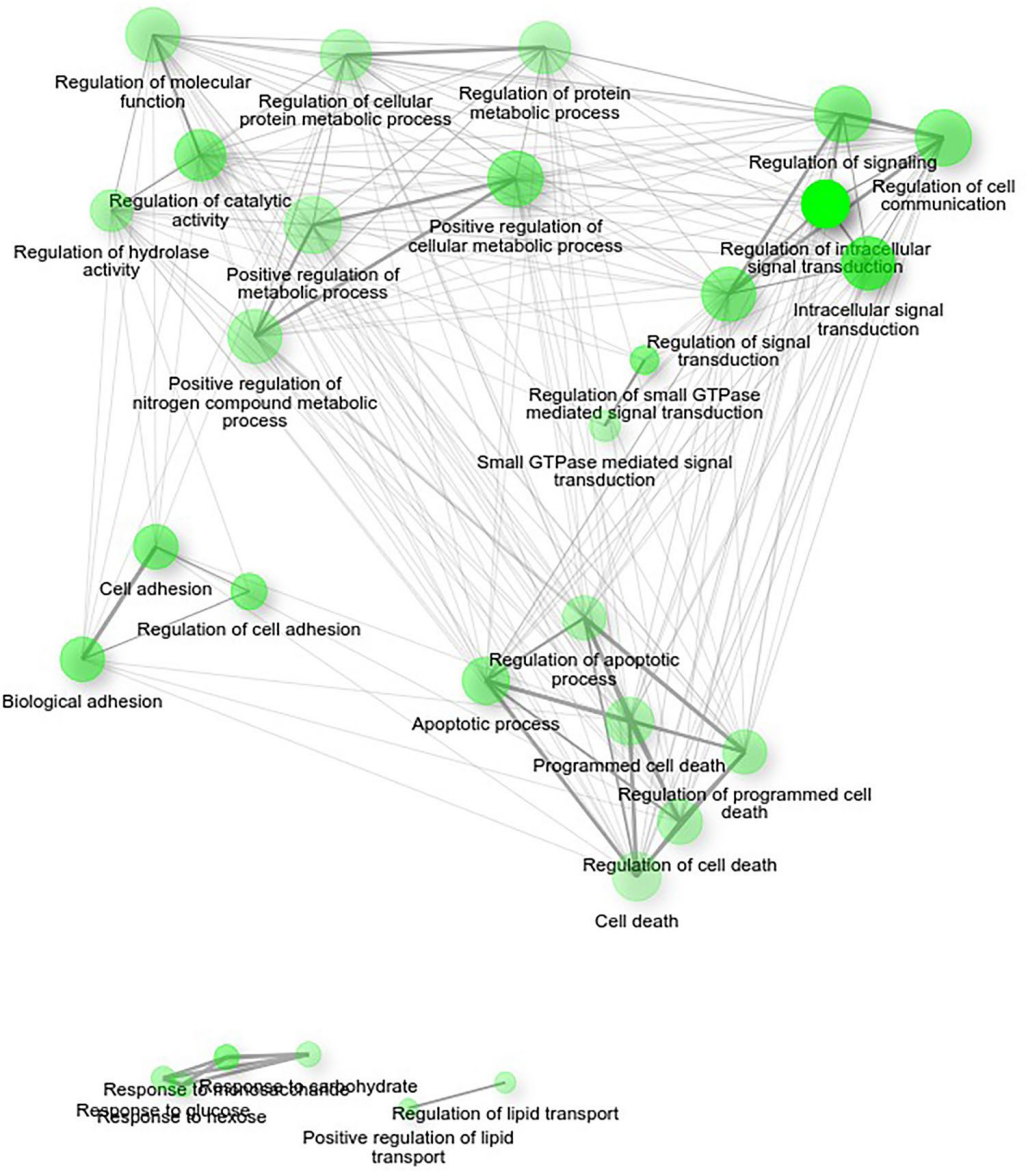
Gene ontology biological processes analysis using ShinyGO algorithm. Picture shows gene networks of differentially expressed genes regulated by ERα in MCF7 constructs which show expression FC > 2 after 24-h estradiol treatment in cells showing low *WWOX* expression relative to high *WWOX* expression

**Fig. 7:**
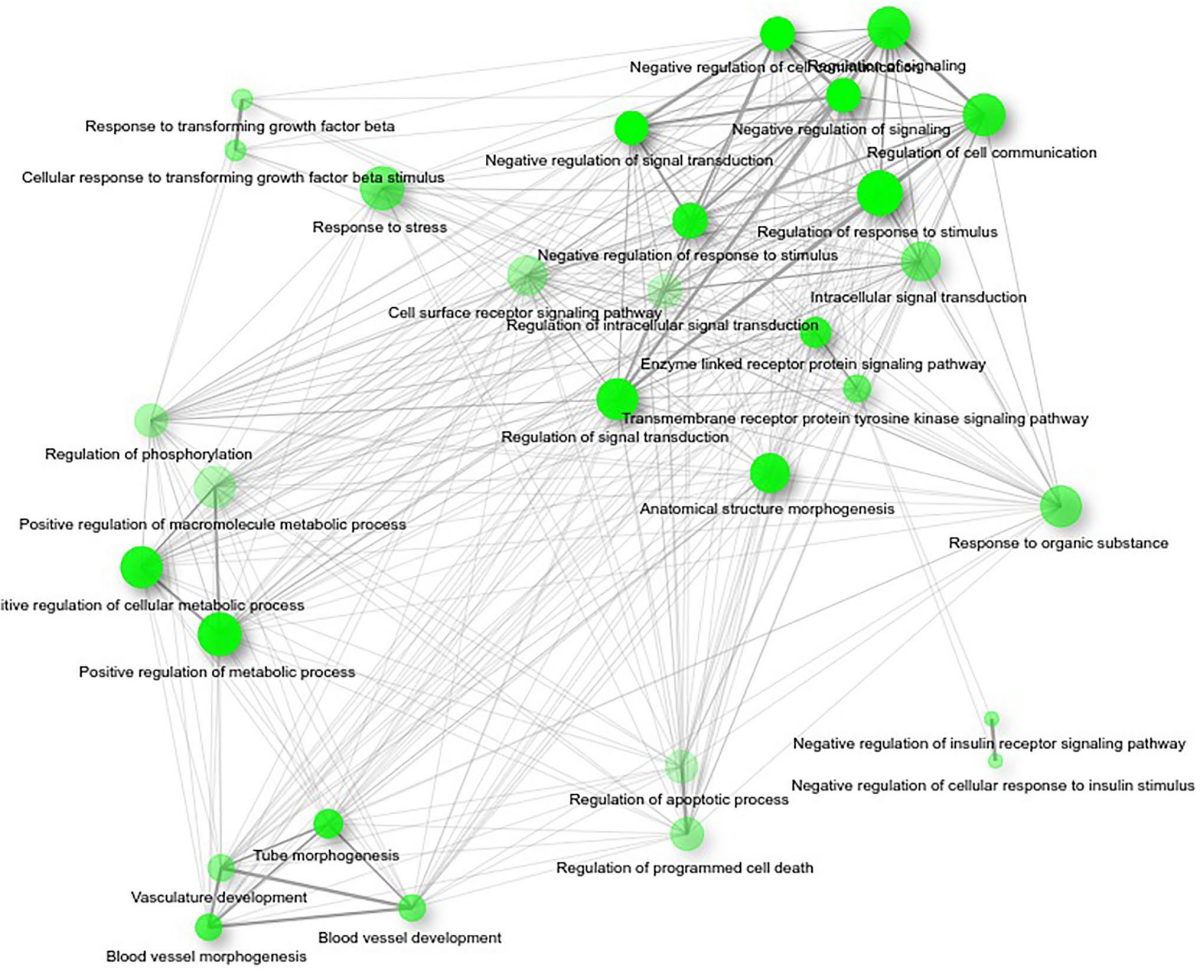
Gene ontology biological processes analysis using ShinyGO algorithm. Picture shows gene networks of differentially expressed genes regulated by ERβ in MDA-MB-231 constructs which show expression FC > 2 after 24-h estradiol treatment in cells showing low *WWOX* expression relative to high *WWOX* expression

The functional analysis encompassed ShinyGO Analysis of ERα targets in MCF7 cell line revealed significant modulation of adhesion-, invasion-, and apoptosis-related genes. To confirm their significant variation, further efforts were focused on gene sets associated with identified processes. Analysis of adhesion-related gene set (GO:0,007,155) comprised of 52 genes, whereas for programmed cell death (GO:0,012,501) pathway 62 genes (list of all identified genes in Supplementary Table 8). Figures 6 and 7 show gene ontology biological processes networks for 30 most significant (FDR < 0.5) pathways of differentially expressed ERα target genes in *WWOX*-silenced MCF7 cells and ERβ target genes in MDA-MB-231 wild-type cells after 24-h estradiol treatment. As shown in both cell lines, major networks are associated with cell death/apoptosis, signal transduction, or EMT and invasion.

### WWOX-ER interplay in adhesion

For adhesion, well diversifying turned out genes encoding laminins (*LAMB3, LAMC1*), myosin-x (*MYO10*), and cadherin 18 (*CDH18*) which are associated with breast cancer invasion and metastasis (Arjonen et al. 2014; Polyak and Weinberg 2009), collagens (*COL9A, COL12A*) claudin-4 (*CLDN4*) was previously found to control proliferation, migration, and apoptosis of MCF7 cells (Ma et al. 2015). Increased expression of mucin *MUC1* has been linked to tumor aggressiveness and autophagy-mediated chemotherapy resistance as reported using the MCF7 cell line model (Garbar et al. 2017).

Interestingly, *AGR2* gene expression was elevated after estradiol treatment in MCF7 and BT20 cell lines with lowered *WWOX*. This anterior gradient 2 protein was found to be a doxorubicin resistance regulator through hypoxia induction by the binding and stabilization of HIF1A protein (Li et al. 2015). *AGR2* expression is induced by estradiol in MCF7 cells with lowered WWOX protein. Moreover, WWOX binds HIF1A in the cytoplasm, therefore regulates its transcriptional activity (Abu-Remaileh and Aqeilan 2014).

Cell adhesion is an important and complex process involved in multiple processes, such as migration, tissue remodeling, and embryogenesis, as well as invasion and metastasis during malignant transformation. All those processes can be modulated by estrogens, particularly in the mammary gland. For instance, in MCF7 cells, 17β-estradiol was found to trigger morphological changes, like a rearrangement of actin filaments and formation of cell–cell and cell-ECM adhesion plaques (DePasquale et al. 1994). There are also numerous associations between adhesion molecules, whose expression was proved significantly altered in tumor cells and estrogen receptors. To give only one example, high expression of E-cadherin is observed in ERα + , low-grade tumors (Parker et al. 2001), whereas P-cadherin is present in ERα − , high-grade tumors (Paredes et al. 2002). There are also associations between adhesion and ERβ, as its loss was found to be related to the reduction of ECM and by decreased levels of the adhesion molecules, such as E-cadherin, or integrin α2 (Forster et al. 2002).

*WWOX* relationship with cell adhesion was already suggested in several studies (Pluciennik et al. 2015; Gourley et al. 2009). Both our NGS results as well as biological experiments confirmed that association, additionally interrelating it with estrogen receptor signaling. Biologically, the most significant changes were observed in adhesion to ECM proteins after estrogen treatment for estrogen-responsive cell lines; however, also *WWOX* up- and downregulation alone resulted in significant alterations in adhesive characteristics (Fig. 1).

### WWOX and TGFα and TGFβ

Epithelial-to-mesenchymal transition in the course of tumor progression is related to extensive morphological changes, enhanced migration, a gain of invasive potential, and loss of adherens junctions. The key event in this process is related to the loss of E-cadherin (Kang and Massague 2004; Cowin et al. 2005). The principal repressor of E-cadherin transcription is *SNAI1* which expression was elevated under estradiol treatment in MCF7 *WWOX*-silenced cells, and also *SNAI2*, *SIP1*, and *TWIST1*. Estrogen interplay can begin, when one of those EMT regulators—*SNAI1*—is repressed by MTA3 (metastatic tumor antigen 3), which is E2 regulated (Fujita et al. 2003). An opposite mechanism was also proposed showing that in non-invasive MCF7 cancer cells, *SNAI1* directly represses expression of ERα expression, thus promoting EMT (Dhasarathy et al. 2007). An analogous mechanism of ERα repression was reported for *SNAI2* in human breast cancer (Bai et al. 2017). ERα can also downregulate EMT through suppression of TGFβ and NF-kB signaling pathways. In the first case, it is achieved through promoting proteasome degradation of Smad2 and Smad3 and in the latter by regulation of NF-kB subunit RelB (Guttilla et al. 2012). Additionally, it was observed that E2 downregulates E-cadherin both in normal and BC epithelial cells (Oesterreich et al. 2003). Also, hypoxia and estrogen signaling may contribute to Notch-mediated EMT (Francesco et al. 2018).

Among numerous cellular processes that *WWOX* plays important role in, there are also reports associating it with EMT in the breast (Li et al. 2018) and endometrial adenocarcinoma (Pluciennik et al. 2015). Our NGS analysis revealed many EMT-related genes well diversifying according to *WWOX* expression, among others, collagens (*COL12A1, COL9A2*—elevated expression in MCF7 *WWOX*-depleted cells under E2); laminins (*LAMC1, LAMB3*) showing lowered expression in MCF7 *WWOX*-depleted with E2; and keratins: *KRT18* which was upregulated in MDA-MB-231 wild-type (low WWOX) under E2 treatment. It is quite interesting because *KRT18* expression is regulated by ERα; therefore, its upregulation in MDA-MB-231 cells is modulated by *WWOX* independently of ERα signaling. Another two keratin genes *KRT8*—which expression is ERα-dependent and *KRT15* ERβ-dependent—were overexpressed in WWOX-depleted MCF7 cells but *KRT15* showed to be downregulated in BT20, respectively. We found that tumor necrosis factor receptor superfamily member 12A (*TNFRSF12A*) was overexpressed under E2 treatment in *WWOX*-depleted MCF7 cells. This protein is involved in tissue remodeling during EMT and was found to be associated with poor survival prognosis in breast cancer patients (Yang et al. 2018). *DUSP6* (overexpressed in MCF7 and BT20 WWOXlow) is associated with brain metastasis in triple-negative breast cancer (Wu et al. 2019).

G0/S2 switch protein (BT20 high expression, MCF7 low expression associated with *WWOX* silencing and E2) mediates the Hippo pathway and induces EMT. Silencing decreased cell proliferation, migration, and invasion of MDA-MB-231 cells (Cho et al. 2019). Another protein *ANXA1* (downregulated in MC7 and BT20 E2 treated and WWOX-depleted cells) which was previously reported to regulate EMT and is associated with highly invasive basal-like breast cancer phenotype (Graauw et al. 2010) was found also to contribute to trastuzumab resistance through AKT activation (Berns et al. 2016; Sonnenblick et al. 2015).

As far as biological experiments are concerned (Fig. 3), no changes in invasive potential were observed for the cell lines, where *WWOX* expression was downregulated (MCF7 and BT20), neither as a result of *WWOX* modulation nor E2 treatment. Estrogen influence on invasiveness was thought observed for T47D cells after estrogen exposure and *WWOX* upregulation. Most significant changes were observed for MDA-MB-231 cell line, where solely *WWOX* gene restoration caused 1.3-fold increase in invasive potential and 1.5-fold increase with simultaneous E2 application.

It was previously reported in several papers that TGFβ signaling is strongly associated with *WWOX* gene expression (Hsu et al. 2016, 2009; Khawaled et al. 2020). In our NGS experiment, we found that TGFβ2 is overexpressed under estradiol treatment in MCF7 WWOX-silenced cells through ERβ (*ESR2*) signaling. As was reported by us and others, another tyrosine receptor kinases pathway which depends on the *WWOX* expression gene is ERBB4 signaling in breast cancer cells (Bednarek et al. 2000, 2001; Nunez et al. 2005; Aqeilan et al. 2005; Iliopoulos et al. 2007).

TGFα-EGFR signaling is associated with carcinogenesis and cancer progression. Interestingly, in our experiment, we identified TGFα elevated expression associated with E2 treatment both in MCF7 and BT20 cell WWOX-depleted cells. TGFα signaling is triggered by its binding to the EGF receptor and then TGFα is cleaved by ADAM17 metalloproteinase which showed stable expression in MCF7 and BT20 cells despite WWOX protein level. There was previously reported that TGFα elevated expression together with ADAM17 expression in breast cancer is associated with lymph node metastasis and worse survival prognosis (Auvinen et al. 1996; Kenny and Bissell 2007).

**Fig. 8:**
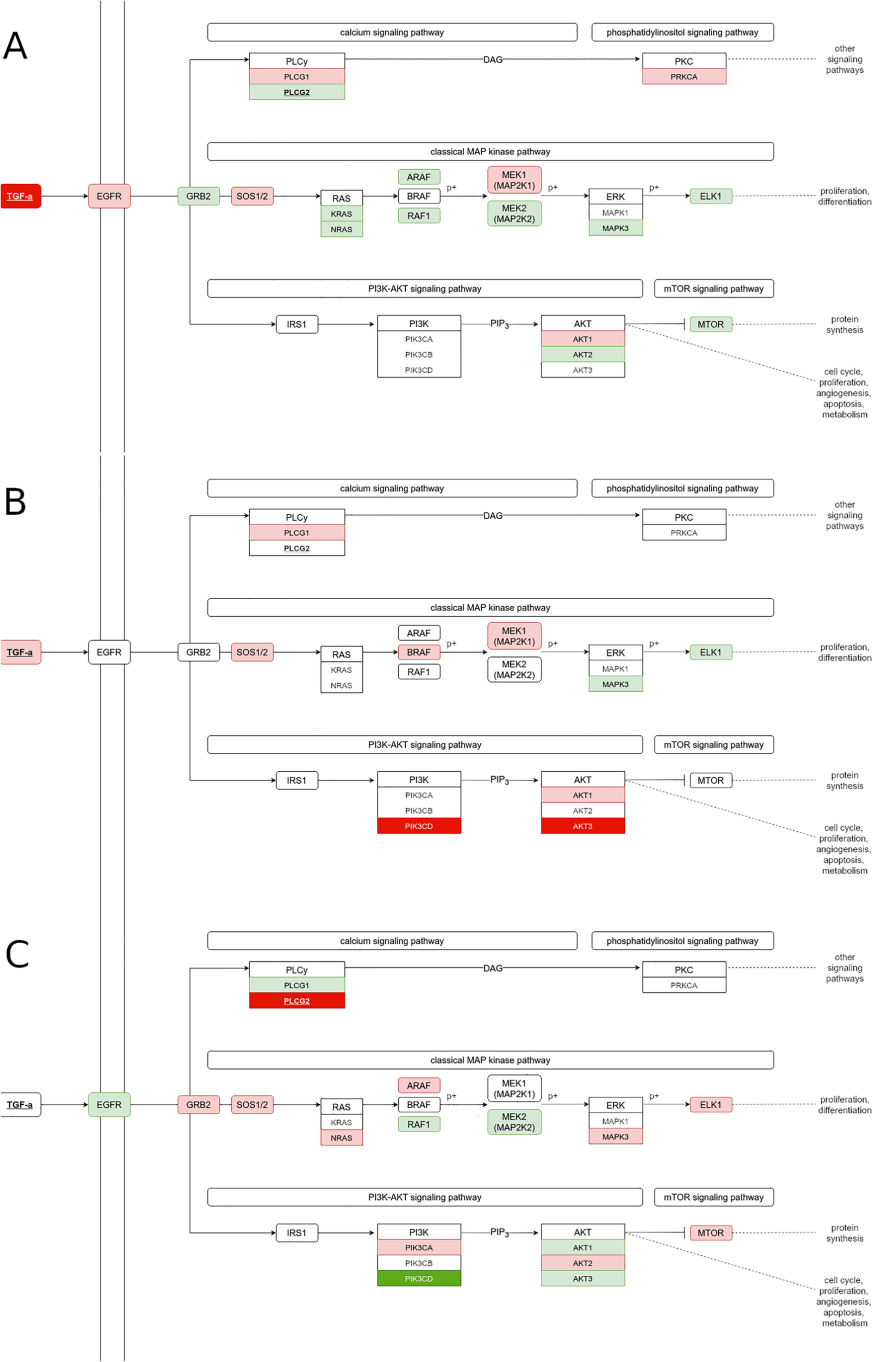
TGFα-EGFR core members of three branches (based on KEGG) colored according to differences in expression in cell line constructs with low versus high expression of WWOX after 24-h estradiol treatment. The red color indicates increase and green color the decrease in expression

Among TGFα-EGFR signaling pathway proteins, some are regulated by estrogen receptors ERα and ERβ. Moreover, we found that several of those proteins expression are also modulated by WWOX. One of them is *STAT5B* which in our experiment shows elevated expression in MCF7 WWOX-silenced cells. It was previously reported that STAT5B ectopically expressed in MCF7 and T47D activates cell proliferation and anchorage-independent growth (Tang et al. 2010). Another protein ERα-regulated protein from TGFα-EGFR is PLCB1 which was recently identified as a metastatic breast cancer driver gene (Mirsadeghi et al. 2021). *PLCB1* was also found to be overexpressed in MCF7 WWOX-silenced cells under estradiol treatment. Other genes from this pathway that we found differentially expressed in our experiments are *CBLC* that was reported as associated with lung cancer progression (Hong et al. 2018) and *CRKL* which promotes tumorigenesis, cancer cell survival, and gefitinib therapy resistance in non-small cell lung cancer (Cheung et al. 2011). We found that expression of *CBLC* is elevated but *CRKL* lowered in WWOX-depleted MCF7 cells after E2 treatment. On another hand, *CRKL* was overexpressed in MDA-MB-231 wild-type cells cultured with estradiol. Figure 8 shows TGFα-EGFR core members of three branches of this signaling pathway, and Table 2 shows the expression differentiation of these genes. Diagrams in Fig. 8 were prepared based on KEGG networks and signaling pathways (KEGG networks TGFA-EGFR-PLCG-PKC N00227, TGFA-EGFR-RAS-ERK N00229, and TGFA-EGFR-PI3K N00231 contributing to KEGG calcium signaling pathway hsa04020, MAP kinase signaling pathway hsa04010, and PI3K-Akt signaling pathway hsa04151, respectively). As shown, all examined breast cancer cell lines show differential expression genes of TGFα-EGFR signaling which is associated with WWOX protein level. Interestingly, *TGFA* expression is regulated by ERα. However, despite estradiol treatment, we found that *TGFA* transcription was highly elevated in MCF7 cells with a low level of WWOX protein. This together with increased expression of *EGFR* in those cells explains the strong tumor-suppressing activity of WWOX in breast cancer. As we see in Fig. 8, MCF7 WWOX-silenced cells also show elevated expression of TGFα-EGFR signaling core members in all three branches of this pathway when compared to MCF7 WT cells with estradiol treatment. A mentioned above, MDA-MB-231 WT cells having low WWOX expression showed lowered TGFβ mRNA level when WWOX was ectopically overexpressed. Moreover, a low level of WWOX in MDA-MB-231 cells is also associated with the TGFα-EGFR pathway several genes elevated expression which is also associated with estradiol treatment. Similar to MDA-MB-231, BT20 breast cancer cells also express variable levels of ERβ receptor (Hanstein et al. 1999). Our analysis depicted in Fig. 8 shows that TGFα-EGFR pathway core gene expression is also affected both by estradiol and WWOX silencing. In all three branches of this pathway, we observed differential expression and some overexpression of *TGFA* under estradiol treatment in WWOX-silenced BT20 cells. To summarize, TGFα-EGFR is regulated in breast cancer mostly by estradiol through ERα regulation of TGFα expression. Our findings showed that such TGFα-EGFR carcinogenic action can be enhanced in breast cancer with low expression of *WWOX* tumor suppressor gene which is supposed to be a very common event in this tumor. As our experiments show, ER-positive MCF7 breast cancer cells showed increased aggressiveness when estradiol action was combined with lowered WWOX protein levels.

**Table 2:** Differences in expression (FC) of genes participating in three major branches of TGFα-EGFR signaling in cell line constructs with low versus high expression of *WWOX* after 24-h estradiol treatment

	BT20	MCF7	MDA-MB-231
TGFA	2.64	100 >	1.16
*EGFR*	1.04	1.38	0.48
	Calcium signaling pathway (KEGG: hsa04020)		
*PLCG1*	1.38	1.24	0.53
*PLCG2*	0.96	0.55	8
*PRKCA*	0.85	1.41	0.8
	MAP kinase signaling pathway (KEGG: hsa04010)		
*GRB2*	1.06	0.68	1.34
*SOS1*	3.16	1.52	0.8
*SOS2*	1.09	0.79	1.24
*KRAS*	0.76	0.65	0.98
*NRAS*	0.7	0.7	1.2
*ARAF*	0.86	0.54	1.46
*BRAF*	1.47	0.75	1.01
*RAF1*	1.29	0.5	0.54
*MAP2K1*	2.02	1.68	0.84
*MAP2K2*	1	0.5	0.67
*MAPK1*	0.98	0.84	0.9
*MAPK3*	0.62	0.32	2.31
*ELK1*	0.65	0.63	2
	PI3K-AKT signaling pathway (KEGG: hsa04151)		
*PIK3CA*	1.06	1.17	1.68
*PIK3CB*	0.74	1.1	0.82
*PIK3CD*	100 >	1	0.16
*AKT1*	2.33	1.73	0.5
*AKT2*	0.85	0.47	1.31
*AKT3*	100 >	1	0.57
*MTOR*	0.83	0.62	1.79

Going downstream of TGFα-EGFR, it was reported that JUNB may enhance EGFR transcription in MCF7 cells (Johnson et al. 2000) and we found this gene to be downregulated in MCF7 WWOX-silenced cells after estradiol stimulation but EGFR expression was elevated anyway in those cells compared to MCF7 with WWOX native level. SHC1 protein (downregulated in MCF7 WWOXlow cells) sequesters ERK inhibiting MAPK/ERK pathway involved in cancer pathogenesis (Lin et al. 2019). Finally, we found the *MYC* gene to be overexpressed in MCF7 and BT20 WWOX-silenced cells under estradiol which connects TGFα-EGFR and TGFβ signaling pathways.

### WWOX and estrogen-mediated apoptosis

Both in normal and malignant mammary epithelial tissue, 17β-estradiol promotes cell proliferation by expression regulation of hormone-responsive genes involved in the cell cycle and/or apoptosis. E2 proved itself to be a potent inhibitor of apoptosis, which regulates the expression of such apoptotic proteins, as Bcl-2 and bclx(L) (Gompel 2000). On the other hand, estrogen was also found to induce apoptosis in BC cells that have been for a long-time deprived of estrogen (LTED cells) or were treated exhaustively with anti-estrogen drugs (Song et al. 2001; Jordan et al. 2005). The pro-apoptotic mechanisms of estradiol action in LTED cells are associated with the death receptors (activation of the complex of Fas death receptor and Fas ligand (FasL) (Gompel 2000)), mitochondrial pathway (release of cytochrome c from the mitochondria and downregulation of Bcl-2 (Lewis et al. 2005; Song et al. 2005)), as well as with repression of the anti-apoptotic factor NF-kB (Lewis-Wambi and Jordan 2009). E2 affects apoptosis also by modulating the c-Jun N-terminal kinase (*JNK*) signaling pathway, which might be additionally ERα-receptor status dependent. Under certain conditions, E2 induced apoptosis and increased phosphorylation of c-jun for ER + MCF7 cells but not in MDA-MB 231 ER − cells (Altiok et al. 2007). Cellular survival might be also mediated by activation of the MAPK and PI3K/Akt signaling pathways via non-genomic action of estrogen-ER complex, which as reported interacts with c-Src protein (Liang and Shang 2013).

Our NGS analysis revealed 60 unique cell death/apoptosis-related genes (programmed cell death GO:0,012,501, apoptotic process GO:0,006,915) well diversifying *WWOX* expression variants, which were estrogen associated (Supplementary Table 8). One of the major apoptosis regulators is BAD pathway which several genes transcription controlled by ERα we found to be overexpressed under E2 treatment in MCF7 *WWOX*-depleted cells. There are *BCL6*, *BCL9L*, and *BMF*, the pro-apoptotic BCL2-modifying factor (Oudenaarden et al. 2018). Downstream in the caspase cascade, the estrogen controlled expression of caspase-4 was induced in WWOX-silenced MCF7 cells. CASP4 gene was previously identified as a major regulator if estrogen induced apoptosis in long-term estrogen-deprived MCF7 cells (Ariazi et al. 2011). Interestingly, despite of *CASP4* expression induction by E2 in MCF7 WWOX-silenced cells, we observed significant reduction of apoptosis. This may be associated with increased expression of anti-apoptotic survivin gene (*BIRC5*) and *JDP2* gene which is another cellular survival protein which represses *AP1* TF and inhibits apoptosis (Lerdrup et al. 2005). Lastly, *WWOX* silencing-depended survival of cancer cells can be also associated with *PRKG2* gene E2-induced expression. *PRKG2* encodes cGMP-dependent protein kinase and regulates broad spectrum of cellular processes such as cell proliferation, differentiation, and cell cycle (Wang et al. 2014; Jiang et al. 2014). Another road of cell death inhibition in MCF7 *WWOX*-silenced cells can lead through Fn14 receptor gene (*TNFRSF12A*) overexpression. Fn4 is the receptor for *TWEAK*. The TNF-like weak inducer of apoptosis and expression of *TNFRSF12A* is controlled by estrogen. It was reported that *TNFRSF12A* ectopically induced expression in MCF7 and T47D cells resulted in a significant induction of invasion and activation of NF-kB signaling (Willis et al. 2008).

There are also some reports suggesting association between *TRADD* and *WWOX*. All thanks to short peptide Zfra (zinc finger-like protein that regulates apoptosis), which regulates TNF-mediated cell death via interacting with TRADD and FADD, as well as JNK1, WWOX, and NF-kB during stress response (Hong et al. 2007; Dudekula et al. 2010). In a proposed model of Zfra/WWOX/TRADD apoptosis, after TNF induction, Zfra physically interacts with TRADD and WWOX and WWOX binds TRADD; therefore, both are recruited to death-inducing signaling complex (DISC) (Dudekula et al. 2010).

Apoptosis is one of numerous cellular processes *WWOX* has been suggested to play roles in Chang et al. (2001); Aqeilan et al. 2004b). Indeed, our NGS results stay in tune with literature reports and all identified apoptosis-related genes were proved to be in tight relationship with *WWOX* and co-regulated by estrogen receptors. Also, our biological experiments (Fig. 4) point at WWOX-ER association with apoptosis. In this case, we can however observe rather the modulation of the apoptosis pathway, which is *WWOX* gene level dependent, than straightforward relationship. Additionally, for both ERα-positive cell lines, estrogen treatment decreased apoptosis, while for estrogen-independent breast cancer cells, increase of apoptosis was noticed. One could suggest that pro-oncogenic activity of E2 is prevalent in that case; however, it is known that oncogenic effect of estrogen is mediated predominantly by ERα activation of cell proliferation-promoting or apoptosis-downregulating genes (Liang and Shang 2013). On the other hand, it was reported that ERα and ERβ can differentially modulate apoptosis-related pathways. Apoptosis-associated JNK kinase was found estrogen activated by ERβ, contrary to ERα, via which this kinase was inhibited (Razandi et al. 1999). This might be the case of our divergent results.

The modulation of adhesion, invasion, and apoptotic properties in all examined breast cancer cell lines, depending on the level of *WWOX* tumor suppressor gene expression, as well as the treatment of estrogen, was confirmed. The obtained results point at a complex role of *WWOX* gene in breast carcinogenesis, which seems to be in tight relationship with the presence of estrogen α and/or β receptors; however, further studies are required to reveal the sophisticated function of the *WWOX* gene.

## Conclusions

As showed in our data and discussed above, *WWOX* in breast cancer modulates significant number of estrogen-regulated genes both ERα- and ERβ-dependent. As our data shows and was reported by others, *WWOX* gene silencing is associated with cancer aggressiveness, epithelial-to-mesenchymal transition, tumor metastasis, and chemoresistance. *WWOX* depletion changes cancer cell proliferation, survival, and apoptosis. We revealed that TGFα-EGFR carcinogenic action is being enhanced in BC cases of low *WWOX* expression which is supposed to be a very common event in this tumor. Additionally, ER-positive MCF7 breast cancer cells showed increased aggressiveness when estradiol action was combined with lowered WWOX protein levels.

## Supplementary Information

Below is the link to the electronic supplementary material.

Supplementary file1 (XLSX 3174 KB)

## Data Availability

The datasets generated for this study can be found in the Gene Expression Omnibus (accession number: GSE140406; https://www.ncbi.nlm.nih.gov/geo/). Supplementary materials supporting the presented findings are available at GitHub repository (https://github.com/orzechmag/wwox-er).
